# Mechanisms by Which Fermented Soybean Meal and Soybean Meal Induced Enteritis in Marine Fish Juvenile Pearl Gentian Grouper

**DOI:** 10.3389/fphys.2021.646853

**Published:** 2021-04-22

**Authors:** Wei Zhang, Beiping Tan, Junming Deng, Xiaohui Dong, Qihui Yang, Shuyan Chi, Hongyu Liu, Shuang Zhang, Shiwei Xie, Haitao Zhang

**Affiliations:** ^1^Laboratory of Aquatic Animal Nutrition and Feed, College of Fisheries, Guangdong Ocean University, Zhanjiang, China; ^2^Aquatic Animals Precision Nutrition and High Efficiency Feed Engineering Research Center of Guangdong Province, Zhanjiang, China; ^3^Key Laboratory of Aquatic, Livestock and Poultry Feed Science and Technology in South China, Ministry of Agriculture, Zhanjiang, China

**Keywords:** *Epinephelus fuscoguttatus* ♀ × *E. lanceolatus* ♂, soybean meal, fermented soybean meal, enteritis, transcriptome sequencing

## Abstract

Soy meals can cause intestinal inflammation and even injury in animals, especially infants and juvenile individuals. This study investigated the effects of fermented soybean meal (FSBM) on the growth and intestinal homeostasis of juvenile pearl gentian grouper and examined the mechanisms by which FSBM and soybean meal (SBM) induced enteritis in fish, using “3+2” full-length transcriptome sequencing. We randomly assigned 720 female juvenile groupers into three treatment groups: FM control group, 20% FSBM group (FSBM20), and FSBM40 group (*n* = 4). Three iso-nitrogenous (50% protein) and iso-lipidic (10% lipid) diets were prepared and fed to fish for 10 weeks. The water volume in each barrel was about 1 m^3^, using natural light and temperature. Results showed that dietary FSBM, at experimental level, significantly affected fish growth and intestinal structure negatively and significantly increased enteritis indices. The degree of intestinal injury and inflammation was determined by the enzyme activities of trypsin and lysozyme, and the contents of IgM, C3, C4, and malondialdehyde, and the expressions of pro-inflammatory genes (*IL1*β, *IL8, IL17*, and *TNF*α) and anti-inflammatory genes (*IL4, IL10*, and *TGF*β*1*). Full-length transcriptome analysis identified 2,305 and 3,462 differentially expressed genes (DEGs) in SBM40 and FSBM40 groups, respectively. However, only 18.98% (920/5,445) of DEGs had similar expression patterns, indicating that high levels of SBM40 and FSBM40 have different metabolic strategies. KEGG enrichment analysis indicated that among the significant pathways, ~45% were related to immune diseases/systems, infectious diseases, and signal transduction in both SBM and FSBM groups. Based on PacBio SMRT sequencing, nine toll-like receptor (TLR) members, including TLR1, TLR2, TLR3, TLR5, TLR8, TLR9, TLR13, TLR21, and TLR22, were detected in intestinal tissues of pearl gentian grouper. TLR-MyD88-NF-κB signaling pathway played an important role in the development of FSBM- and SBM-induced enteritis in pearl gentian grouper; however, TLR receptors used in SBM and FSBM groups were different. TLR1, TLR8, TLR13, and TLR22 were the main receptors used in FSBM group, while TLR5, TLR8, TLR9, TLR21, and TLR22 were the main receptors used in SBM group. Present study provides valuable theoretical references for further research on soy protein-induced enteritis in fish.

## Introduction

Fish meal (FM) is an important protein source in aquaculture; however, due to the expansion of aquaculture industry and scarcity of fish resources, there has been an increase in the price of fish meal in recent years. Therefore, it is necessary to find low-cost and high-availability protein source alternatives to FM (Gu et al., 2018). Soybean meal (SBM) is widely studied as a promising substitute for fish meal because of its moderate price, balanced amino acid profile, and availability (Hedrera et al., 2013). However, SBM contains several anti-nutritional factors (ANFs), such as protease inhibitors, oligosaccharides, lectins, phytic acids, and saponins, which can limit its use in aquaculture (Mosberian-Tanha et al., 2016). Fermented soybean meal (FSBM) is a processed product of SBM; the fermentation process aims to improve the quality of soybean meal, reduce ANFs, and increase soluble protein and small peptide contents (Kader et al., 2012). Presently, quality standards for FSBM have been formulated in China, but there are still a few problems. The fermentation process only reduces the ANF contents of soybean meal, but do not completely remove the ANFs (Hong et al., 2005).

SBM is a commonly used protein to substitute for FM in aquatic feed, and previous studies on soy proteins mainly focused on its effects on fish growth. Different kinds of fish have different adaptabilities to soy protein replacement. Ai and Xie (2002) reported that 39% replacement of FM with SBM in the diets of *Silurus meridionalis* had no significant effect on growth. An et al. (2018) found that when the substitution level of FM with SBM exceeded 20%, the growth performance, feed conversion ratio, and digestive enzyme activities of *Epinephelus coioides* were significantly negatively affected. Zhou et al. (2014) reported that a replacement of over 30% FM with soybean meal not only reduced the growth performance and feed efficiency of *E. coioides* but also decreased its immunity. A previous study reported that 14% inclusion of FSBM had no significant effects on the weight gain rate and specific growth rate of *E. coioides*; however, these indicators decreased significantly with an increase in dietary FSBM, and the optimal level of FSBM substitution for FM (52% basic fish meal) was 10% (Luo et al., 2004). Related studies have also shown that high levels of FSBM inclusion can alter intestinal flora homeostasis, hinder probiotic growth, and may cause intestinal infection (Xu et al., 2013). Generally, when the FM in diets is substituted with soy protein in excess, especially in carnivorous fish, there is a significant negative impact on fish intestinal health, such as changes in intestinal morphology, enteritis, reduced activities of intestinal digestive enzymes, and altered digestion and absorption processes.

Previous studies on soy meal-induced enteritis (SBMIE) in fish mainly concentrated on related enzyme activities, gene expression, and on the effects of single ANFs on fish enteritis. However, studies have indicated that SBMIE in fish may be induced by the combined effects of multiple ANFs (Sahlmann et al., 2013; Gajardo et al., 2016). Our previous research also supported this result (Zhang et al., 2019). At the transcriptome level, studies on SBMIE used mainly DNA microarray technology (Kortner et al., 2012), but there are few reports on the applications of second- or third-generation transcriptome sequencing technology with higher sequencing flux and accuracy. The full-length transcriptome is based on the third-generation sequencing platform of PacBio Sequel, which can directly obtain the complete transcripts containing the 5′UTR, 3′UTR, and polyA tail without splicing. Moreover, it can perform transcript specific expression analysis with the help of second-generation sequencing data to obtain more comprehensive annotation information. The analysis of full-length transcriptome PacBio SMRT sequencing data of the intestines of pearl gentian grouper fed different levels of soy meal has been reported in our previous study (Zhang et al., 2021b). Currently, the mechanism of SBMIE in fish is unclear and lacks a systematic and comprehensive explanation. Therefore, it is necessary to use new omics technology to carry out further research under the condition of whole soybean meal substitution.

The pearl gentian grouper *Epinephelus fuscoguttatus* ♀ × *Epinephelus lanceolatus* ♂ is a typical marine carnivorous fish, with fast growth rate, high market value, and high disease resistance. It is widely farmed in China and all over the world (Liu et al., 2018). Our previous research showed that high substitution levels of soy meal, such as SBM and FSBM, induced enteritis in the pearl gentian grouper. Additionally, our unpublished research investigated the characteristics of SBM-induced enteritis in the pearl gentian grouper. The present study aimed to evaluate the effects of FSBM on the growth and physiology of pearl gentian grouper and compare the mechanisms by which FSBM and SBM induce enteritis at the transcriptome level. The findings of this study may serve as a reference for solving fish intestinal health issues caused by substituting fish meal with plant proteins.

## Materials and Methods

### Experimental Diets

The composition and chemical analysis of the experimental diets used in the present study are shown in Table 1, and the formula of SBM diets is shown in Supplementary Table 1. We procured red fish meal (72.53% crude protein and 8.82% crude lipid) from Corporación Pesquera Inca S.A.C., Bayovar Plant, Peru. The FSBM, which was fermented using *Bacillus*, was provided by Xijie Foshan Co. Ltd (Foshan, China). The SBM was provided by Zhang Haibao Feed Co., Ltd. (Zhanjiang, China). We formulated three iso-nitrogenous (~50% crude protein) and iso-lipidic (10% total lipid) diets. The three experimental diets (FM (control), FSBM20, and FSBM40) were formulated by replacing 0, 20, and 40% of FM protein with FSBM protein, respectively. Lysine and methionine were added to the experimental diets to balance for amino acids (Miao et al., 2018). The detailed preparation process and storage conditions of the experimental diets were described in our previously published work (Zhang et al., 2019). The SBM diets were prepared in the same method, which named SBM20 and SBM40. The amino acid contents of FSBM and SBM diets are shown in Supplementary Table 2. The contents of ANFs in FSBM and SBM diets are shown in Supplementary Table 3. The ANFs were detected using high performance liquid chromatography (HPLC) according to the Chinese national standard GB/T23788-2009 and GB/T16631-2008, and Soyasaponin I was detected by HPLC-ESI/MS^2^.

**Table 1:** Formulation and proximate composition of the experimental diets (%, dry matter).

**Ingredients (%)**	**Diets**		
	**FM**	**FSBM20**	**FSBM40**
Red fish meal	50.00	40.00	30.00
Fermented soybean meal	0.00	11.94	23.89
Vital wheat gluten	5.00	5.00	5.00
Wheat flour	18.00	18.00	18.00
Casein	4.60	4.60	4.60
Gelatin	1.00	1.00	1.00
Fish oil	3.02	3.76	4.49
Soybean oil	2.00	2.00	2.00
Soybean lecithin	2.00	2.00	2.00
Microcrystalline cellulose	11.48	8.67	5.84
Calcium monophosphate	1.50	1.50	1.50
Ascorbic acid	0.05	0.05	0.05
Choline chloride	0.50	0.50	0.50
Vitamin premix^a^	0.30	0.30	0.30
Mineral premix^b^	0.50	0.50	0.50
Ethoxyquin	0.05	0.05	0.05
Lysine^c^	0.00	0.06	0.13
Methionine^c^	0.00	0.07	0.15
**Proximate composition (%, dry matter)**
Crude protein	50.97	50.82	50.45
Crude lipid	10.15	10.38	10.54

a *Vitamin premix consisted of (g/kg premix): VB_1_ 17.00 g, VB_2_ 16.67 g, VB_6_ 33.33 g, VB_12_ 0.07 g, VK 3.33 g, VE 66.00 g, retinyl acetate 6.67 g, VD 33.33 g, nicotinic acid 67.33 g, D-calcium pantothenate 40.67 g, biotin 16.67 g, folic acid 4.17 g, inositol 102.04 g, and cellulose 592.72 g*.

b *Mineral premix consisted of (g/kg premix): FeSO_4_·H_2_O 18.785 g. ZnSO_4_·H_2_O 32.0991 g, MgSO_4_·H_2_O 65.1927 g, CuSO_5_·5H_2_O 11.0721 g, CoCl_2_·6H_2_O (10%) 5.5555 g, KIO_3_ 0.0213 g, KCl 22.7411 g, Na_2_SeO_3_ (10%) 0.5555 g, zeolite powder 843.9777 g*.

c *Lysine and Methionine were added to balance amino acid with FM control group*.

### Feeding Trial and Breeding Management

Healthy juvenile groupers (100% female), with an average weight of ~9 g, were purchased from Zhanjiang city, China. The fish were domesticated under experimental conditions for 1 week and fed commercial diets (Haida Aquatic Feed Co., Ltd., Zhanjiang, China). Before the experiment, the fish were fasted for 24 h and grouped after being anesthetized with eugenol (1:10,000). Thereafter, 60 fish of similar sizes were randomly distributed into a 1,000 L cylindrical fiberglass tank. Each experimental group consisted of four replicates and each group was fed twice daily at 8:00 and 16:00, until apparent satiation, for 10 weeks. Feed consumption was determined and recorded following the procedure described by Zhang et al. (2019). The experiment was carried out in the indoor culture system of the Zhanjiang Marine Biological Research Base, China. The tanks were continuously aerated with air stone. Throughout the study period, the tanks were illuminated by sunlight, temperature was maintained at 29 ± 1°C, ammonia and nitrate remained below 0.03 mg L^−1^, and dissolved oxygen was never <7 mg L^−1^. In the first 2 weeks, the daily water exchange rate was 60% in each barrel, but increased to ~100% subsequently.

### Sample Collection

At the end of the feeding trial, the fish were starved for 24 h before sampling. Thereafter, fish in each tank were counted and weighed to determine the weight gain rate (WGR), specific growth rate (SGR), feed conversion ratio (FCR), and survival rate (SR). The final body weight (FBW) and hepatosomatic index (HSI) of fish were calculated.

For enzyme activity analysis, six fish were randomly collected from each tank. After anesthesia with eugenol (1:10,000), blood was drawn from the caudal vein and stored at 4°C overnight. Thereafter, serum was separated from the blood samples by centrifugation at 3,500 rmin^−1^ for 10 min. The serum was stored at −80°C for enzyme activity analysis. Subsequently, the distal intestine (DI) and liver of fish were removed, cleaned of any mesenteric and adipose tissue, and washed with deionized water. Portions of the DI and liver samples from each fish were placed into tubes, frozen in liquid nitrogen, and stored at −80°C for enzyme activity analyses. Part of the DI samples was cut into pieces, placed in a tube containing RNAlater overnight at 4°C, and stored at −80°C for gene expression analysis.

For transcriptome sequencing, DI samples were taken from four fish per tank and treated as described above. Thereafter, the DI samples were placed in tubes, frozen in liquid nitrogen, and stored at −80°C for transcriptome sequencing.

### Histological Observation of Enteritis

For histological evaluation, DI samples were collected from three fish per tank. DI tissue samples of appropriate length were stored in a 4% paraformaldehyde universal tissue fixative (Servieobio Technology Co., Ltd., Wuhan, China) for 24 h until further hematoxylin and eosin (H&E) staining.

The H&E-stained sections were examined with optical microscopy (Olympus CKX41 microscope, Tokyo, Japan). The degree of SBMIE in the DI tissues of the pearl gentian grouper was assessed based on the following: (1) the appearance and length of mucosal plica; (2) the degree of widening of the lamina propria in the mucosal plica; (3) the number and size of vacuoles in the nucleus; and (4) the thickness of connective tissue between the base of the mucosal fold and dense layer. The score of each indicator ranged from 1 to 5. “1–2” indicated normal morphological features, “3” indicated obvious morphological features of enteritis, and “5” indicated severe symptoms of enteritis. The detailed scoring criteria for H&E sections of the different indicators are shown in Supplementary Table 4. The score was analyzed via analysis of variance (ANOVA) using SPSS 22.0 (SPSS Inc., Chicago, IL, USA).

### Analysis of Biochemical Indicators

The BCA method (Beyotime Biotechnology Co., Ltd, Shanghai, China) was used to determine the protein contents of the intestine and liver samples collected from the fish. Furthermore, we determined the trypsin activity, concentrations of immunoglobulin M (IgM), complement 3 (C3), complement 4 (C4), and malondialdehyde (MDA) in the DI tissues, and serum lysozyme (LYS) activity using fish ELISA kits (Shanghai Jianglai Biotechnology Co., Ltd., Shanghai, China) according to the manufacturer's instructions.

### Immune-Related Gene Expression

The total RNA of intestinal tissue was extracted using the Trizol kit (Invitrogen, Carlsbad, CA, USA) according to the manufacturer's instructions. RNA sample integrity was determined using 1% agarose. The RNA samples were stored at −20°C until further use. Synthesis of first-strand cDNA was performed using the Evo M-MLV reverse transcription kit (Takara, Japan). The primers used in this study were synthesized by Shenggong Bioengineering Co., Ltd. (Shanghai, China), and designed using the Primer Premier 5.0 software (Premier Biosoft International, Palo Alto, CA). The sequence primers used in this study were obtained from the third-generation transcriptome sequence database of DI tissues of pearl gentian grouper in our lab. The primers for pro-inflammatory (*IL1*β, *IL8, IL17, TNF*α, and *CSF1*) and anti-inflammatory genes (*IL4, IL10, TGF*β*1*, and *hepcidin*) are listed in Table 2. The internal reference gene was β-actin. The expression levels of these genes were detected with qRT-PCR (Mastercycler ep realplex, Eppendorf, Germany). The PCR reaction conditions were as follows: 95°C for 2 min, 1 cycle; 95°C for 15 s, 60°C for 10 s, 72°C for 20 s, and 40 cycles. Expression of the target genes was determined using the 2^−Δ*ΔCT*^ method (Livak and Schmittgen, 2001).

**Table 2:** PCR primers for intestinal immune-related genes of pearl gentian grouper.

**Gene**	**Forward5^**′**^-3^**′**^**	**Revise3^**′**^-5^**′**^**	**Size (bp)**
*IL1β*	AAGGTGGACGCCAACAGACA	GTTCACTGCAGGCTCAGGGA	153
*IL8*	TGTGGCACTCCTGGTTCTCC	GGGTTCACCTCCACCTGTCC	132
*IL17*	GAGAGGACGGTGTCTGTGTGG	CATGCACAGTTGAGGGTGTGG	101
*TNFα*	AACTGTGTGTCCCCACTGCC	CCACAGATGGCCCAGGTCAT	81
*CSF1*	CCAAGATGGCCTCCTACGCA	CAGCAGTGAGGAGGGTCTGG	134
*IL4*	GCAGTGAGTGAAGCCATCGC	TGCAGTTCCTGATAGCGCGA	146
*IL10*	ACACAGCGCTGCTAGACGAG	TAGACTTGTGCCACGACGGG	142
*TGFβ1*	CTTCTCCTCCTCCTCGCTGC	GATGTTGCTGAGGGCTTCGC	195
*Hepcidin*	TGTCAATGACCCACTGAG	TCCACTGCAAACTGCTGGGC	105
	CCTCG		
*β-actin*	GGCTACTCCTTCACCACCACA	TCTCCAAGGCAACGGGTCT	188

### “3+2” Transcriptome Sequencing

The detailed process of the “3+2” full-length transcriptome sequencing was described by (Zhang et al., 2021b). Briefly, qualified RNA was first obtained for sequence library construction. For third-generation library construction and sequencing, polyA mRNA was enriched using magnetic beads containing Oligo (dT), and then the mRNA was reverse transcribed into cDNA. After PCR amplification, cDNAs with fragments larger than 4 kb were screened and amplified again to obtain enough cDNA. The full-length cDNA was repaired to construct the equal-mole hybrid library containing non-screening fragments and fragments larger than 4 kb. The sequences without joints at both ends of the cDNA were removed; then, the complete SMRT bell library was constructed by combining primers and binding DNA polymerase. Finally, after quality inspection, the library was sequenced using the PacBio sequence platform. For second-generation library construction and sequencing, polyA mRNA was enriched using magnetic beads containing Oligo (dT), and the fragment buffer was added to make it into short fragments. The short mRNA was used as a template to synthesize cDNA. Terminal repair, polyA addition, and sequencing adaptor were performed. Subsequently, the target fragments were recovered for PCR amplification to complete the preparation of the whole library. Finally, the libraries were sequenced using the Illumina HiSeqTM 4000.

After the sequence was completed, the Illumina RNAseq sequencing data was used to correct the PacBio SMRT sequencing results to obtain high-quality sequences for subsequent analysis. The raw reads of the PacBio SMRT and Illumina sequencing were deposited in the NCBI Sequence Read Archive (SRA) with accession numbers PRJNA664623 and PRJNA664416, respectively.

### Analysis of the Differential Genes (DEGs)

The present study was conducted to compare the mechanisms by which high levels of FSBM and SBM (FSBM40 and SBM40) diets induce enteritis in juvenile pearl gentian grouper. Firstly, we identified DEGs in the DI tissues of pearl gentian grouper in the FSBM40 and SBM40 groups. The screening threshold was |log_2_FC| > 1 and *P* < 0.05. Genes meeting the above conditions were identified as DEGs. We constructed a Venn diagram of the DEGs identified in the FSBM40 and SBM40 groups, indicating DEGs that were common to both groups and unique to each group. Finally, gene ontology (GO) function annotation and Kyoto Encyclopedia of Genes and Genomes (KEGG) pathway enrichment analyses were carried out for the DEGs to identify genes involved in signal pathways related to immune diseases/systems, infectious diseases, and signal transduction. Genes were considered significantly enriched at *P* < 0.05.

### Validation of Quantitative Real-Time PCR

To validate the accuracy of “3+2” transcriptome sequencing, 14 genes related to the development of inflammation were selected, including *TLR1, TLR2, TLR3, TLR5, TLR8, TLR9, TLR13, TLR21, TLR22, MyD88, IKK*α, *IKK*β, *I*κ*B*α, and *p65* for qRT-PCR. The primer design, synthesis, and template source of all the genes were the same as those mentioned in section Analysis of biochemical indicators. The internal control gene was β*-*actin (Table 3). Expression of the genes was detected using qRT-PCR (Mastercycler ep realplex, Eppendorf, Germany). The PCR reaction conditions were follows: 1 cycle at 95°C for 2 min, followed by 40 cycles at 95°C for 15 s, 60°C annealing for 10 s, and 72°C for 20 s. All reactions were performed in triplicate. Melting curve analysis was performed to determine target specificity. The qRT-PCR results were calculated using the 2^−ΔΔCT^ method (Livak and Schmittgen, 2001).

**Table 3:** Primers used for real-time quantitative PCR analysis.

**Gene**	**Forward5^**′**^-3^**′**^**	**Revise3^**′**^-5^**′**^**	**Size (bp)**
*TLR1*	CCAGGTAGGTGAGGTGGCAG	GAGAGCCAGAAGGTGCTGCT	175
*TLR2*	TCTGCAAGCTGCGAAGAGTC	CCAGAACCTGGGAATCTGGC	80
*TLR3*	CTTCTCGTCTCGGCGGTGAT	GACCGCACTAAGGCTGAGGT	135
*TLR5*	GGTGGTAGGGAAGGTGGCTC	TGTCAGACTGTGCAGCGTCA	200
*TLR8*	CGTGGATGTGATCGTGCTGC	CTGTTCTGGGCCACTCCACA	110
*TLR9*	GCAGCGACTTCTGGACGAGA	CTCGGCCAGGACAACACAGA	126
*TLR13*	CCATCCCGACCATCACTCGT	CTCCCATCGGTGCATGCAAC	132
*TLR21*	GGACGTTCTCCTGCTCGTGT	AACAGCTCTTGGGCCTGTGT	147
*TLR22*	GGACCTTCAACTCCTCAC	GTTGGGATGCTGCAGG	94
	TGACG	AGATG	
*MyD88*	GCATCTTGCGCTTCCTCACC	CCTGGTCCTTGGTTACGGCA	107
*IKKα*	GCCAGCAGCACATCACTTCC	GGTTCTGGAGGTCTACGGCC	186
*IKKβ*	GCCTTGGAGCCTCATGGACT	CGGTTTGGACGAAGCGGATG	169
*IκBα*	ATGCAAAGGAGCAGCGTAACG	GAGGTTGGGGTCTGCTCCT	107
*p65*	TCAACCCAGTCCAAGCAGCA	GATGCTGCCAGCTGAACGTC	107
*β-actin*	GGCTACTCCTTCACCACCACA	TCTCCAAGGCAACGGGTCT	188

### Statistical Analysis

Growth performance was calculated using the following formulas:

$$\begin{array}{l} \text{ Weight gain rate }(\text{WGR},\;\%)=\frac{\text{100 }\times \;(\text{final body weight - initial body weight})}{\text{initial body weight}}\\ \text{Specific growth rate }(\text{SGR},\;\%)=\frac{\begin{array}{c} 100\;\times \;[Ln\;(\text{final body weight})\\ -\;Ln\;(\text{initial body weight})] \end{array}}{\text{days}}\\ \text{ Feed conversion ratio }(\text{FCR})=\frac{\text{feed intake}}{\left(\text{final body weight - initial weight}\right)}\\ \text{ Hepatosomatic index }\\ \;\left(\text{HSI},\;\%\right)=\;100\;\times \;(\frac{\text{hepatic weight}}{\text{body weight}})\\ \text{ Survival rate }\left(\text{SR},\;\%\right)=100\;\times \;(\frac{\text{final fish number}}{\text{initial fish number}}) \end{array}$$

The results were presented as mean ± S.D. Statistical analysis was conducted using SPSS 22.0 (SPSS Inc., Chicago, IL, USA). All data were analyzed using one-way ANOVA after the homogeneity variance test. *P* < 0.05 was accepted as statistically different.

## Results

### Growth Performance

The results showed that the FSBM20 and FSBM40 groups had significantly (*P* < 0.05) lower WGR and SGR values compared than those of the control group (Table 4). FCR increased significantly (*P* < 0.05) in the FSBM20 and FSBM40 groups compared with that in the control group. HSI and SR were not significantly (*P* > 0.05) affected by FSBM supplementation. The growth performance of SBM20 and SBM40 groups exhibited the similar results in Supplementary Table 5.

**Table 4:** Effect of different levels of fermented soybean meal substitute for fish meal protein on the growth of pearl gentian grouper (*n* = 3).

**Parameters**	**FM**	**FSBM20**	**FSBM40**
IBW (g)	12.55 ± 0.00	12.55 ± 0.06	12.55 ± 0.04
WGR (%)	485.14 ± 7.08^a^	454.27 ± 14.70^b^	427.22 ± 7.28^c^
SGR (%d)	2.60 ± 0.02^a^	2.52 ± 0.04^b^	2.44 ± 0.02^c^
FCR	0.84 ± 0.01^a^	0.89 ± 0.03^b^	0.95 ± 0.02^c^
HSI (%)	2.43 ± 0.45	2.38 ± 0.32	2.19 ± 0.19
SR (%)	99.17 ± 0.96	99.17 ± 1.67	99.58 ± 0.84

*Different superscripted letters within each line indicate significant differences (P < 0.05)*.

### Histological Evaluation

**Figure 1:**
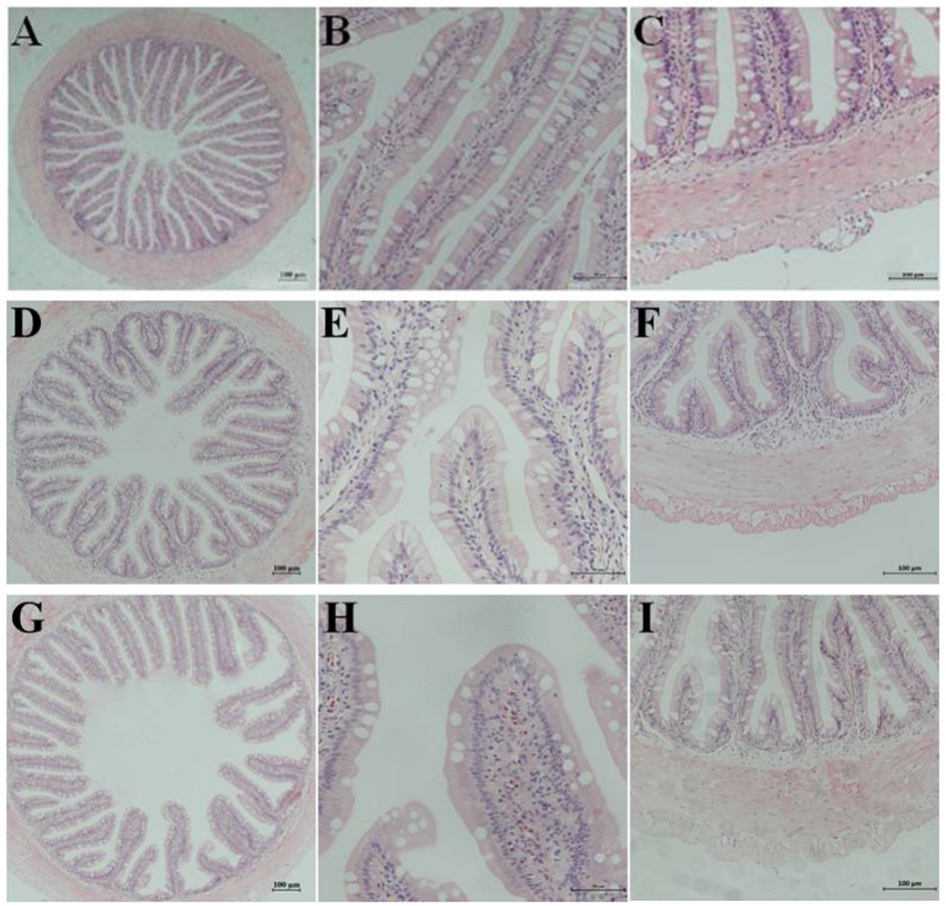
Hematoxylin-eosin staining in the hindgut of pearl gentian grouper. Representative histomorphological images from hematoxylin and eosin stain ed sections of distal intestine of grouper juvenile depicting the inflammatory changes in grouper fed the FM **(A–C)**, FSBM20 **(D–F)**, and FSBM40 **(G–I)** diets. **(A,D,G)** Representative images of decreased height and increased fusion of the mucosal folds in FSB M20 and FSBM40 group. **(B,E,H)** Representative images of increased width and cellular (leucocyte) infiltration (asterisk) of the lamina propria (arrows) in FSBM20 and FSBM40 group. **(C,F,I)** Representative images of increased width and cellular (leucocyte) infiltration of the submucosa. FM, fish meal control group; FSBM20, 20% FSBM protein replacement level to FM protein; FSBM40, 40% FSBM protein replacement level to FM protein. Arrow lamina propria; asterisk inflammatory infiltration.

The semi-quantitative analysis of H&E staining showed that the different substitution levels of FSBM caused DI enteritis in the pearl gentian grouper. FSBM significantly (*P* < 0.05) influenced the enteritis indices of mucosal folds, lamina propria, supranuclear vacuoles, and connective tissue of fish DI intestine. Compared with the FM control group, the FSBM groups showed significantly (*P* < 0.05) higher enteritis indices. The degree of enteritis in the FSBM40 group was the most serious, followed by that of the FSBM20 group (Figure 1 and Table 5).

**Table 5:** Semi-quantitative histological evaluation of intestinal sections (*n* = 10).

**Parameters**	**FM**	**FSBM20**	**FSBM40**
Mucosal folds	1.27 ± 0.12^a^	3.10 ± 0.20^b^	4.17 ± 0.55^c^
Lamina propria	1.47 ± 0.35^a^	3.83 ± 0.31^b^	4.50 ± 0.10^c^
Supranuclear vacuoles	1.27 ± 0.21^a^	3.13 ± 0.25^b^	4.13 ± 0.35^c^
Connective tissue	1.47 ± 0.25^a^	2.43 ± 0.61^b^	4.30 ± 0.46^c^

*Different superscripted letters within each line indicate significant differences (P < 0.05)*.

### Determination of Biochemical Indices

Trypsin activity was significantly (*P* < 0.05) higher in the intestinal tissues of fish in the FSMB20 and FSMB40 groups compared with that in the tissues of fish in the control group (Table 6). Similarly, IgM, C3, C4, and MDA concentrations increased significantly (*P* < 0.05) with an increase in dietary FSBM (*P* < 0.05). Additionally, serum LYS concentration increased significantly (*P* < 0.05) with an increase in dietary FSBM addition.

**Table 6:** Effect of different levels of fermented soybean meal substitute for fish meal protein on the enzyme activities of pearl gentian grouper (*n* = 3).

**Parameters**	**FM**	**FSBM20**	**FSBM40**
Trypsin (U/mg)	597.33 ± 75.53^a^	1046.67 ± 76.95^b^	1194.50 ± 90.84^b^
IgM (μg/mg)	94.33 ± 4.22^a^	66.40 ± 6.32^b^	50.84 ± 5.60^c^
C3 (μg/mg)	85.58 ± 5.31^a^	62.71 ± 8.35^b^	51.34 ± 7.73^b^
C4 (μg/mg)	128.83 ± 10.17^a^	91.40 ± 7.87^b^	76.83 ± 8.28^c^
MDA (nmol/mg)	2.49 ± 0.41^a^	3.44 ± 0.43^b^	4.15 ± 0.46^c^
LYS (U/g)	5.45 ± 0.47^a^	4.01 ± 0.51^b^	4.13 ± 0.86^b^

*Different superscripted letters within each line indicate significant differences (P < 0.05)*.

### Immune-Related Gene Expression

The results of gene expression analysis showed that the expression of pro-inflammatory genes, *IL8, IL17, TNF*α, and *CSF1* was significantly (*P* > 0.05) higher in the FSBM40 group than that in the control group. However, the levels of expression of these genes were not significantly (*P* > 0.05) different between the FSBM20 and control groups. Expression of the pro-inflammatory gene *IL1*β was significantly (*P* < 0.05) higher in the FSBM20 and FSBM40 groups (Table 7).

**Table 7:** Effect of fermented soybean meal substitute for fish meal protein on the pro-inflammatory gene expression in hindgut of pearl gentian grouper (*n* = 3).

**Gene**	**FM**	**FSBM20**	**FSBM40**
*IL1β*	1.16 ± 0.16^a^	1.31 ± 0.03^b^	1.74 ± 0.23^c^
*IL8*	1.00 ± 0.08^a^	1.02 ± 0.21^a^	1.62 ± 0.04^b^
*IL17*	1.00 ± 0.07^a^	0.94 ± 0.12^a^	1.35 ± 0.01^b^
*TNFα*	1.01 ± 0.15^a^	1.12 ± 0.23^a^	4.09 ± 0.61^b^
*CSF1*	1.01 ± 0.13^a^	1.12 ± 0.21^a^	3.13 ± 0.26^b^

*Different superscripted letters within each line indicate significant differences (P < 0.05)*.

Furthermore, the expression of anti-inflammatory genes *IL4, IL10*, and *TGF*β*1* was significantly (*P* < 0.05) lower in the FSBM40 group than that in the control group. However, the expression of anti-inflammatory genes in the FSBM20 and control groups was not significantly different, except the expression of *IL10*, which was significantly (*P* < 0.05) lower in the FSBM20 group than that in the control group. Expression of the antimicrobial peptide *hepcidin* increased significantly (*P* < 0.05) in the FSBM40 group compared with that in the control group (Table 8).

**Table 8:** Effect of fermented soybean meal substitute for fish meal protein on the anti-inflammatory gene expression in hindgut of pearl gentian grouper (*n* = 3).

**Gene**	**FM**	**FSBM20**	**FSBM40**
*IL4*	1.01 ± 0.15^a^	0.94 ± 0.21^a^	0.48 ± 0.06^b^
*IL10*	1.02 ± 0.05^a^	0.63 ± 0.06^b^	0.56 ± 0.03^b^
*TGFβ1*	1.00 ± 0.08^a^	0.98 ± 0.09^a^	0.13 ± 0.01^b^
*Hepcidin*	1.02 ± 0.25^a^	0.92 ± 0.11^a^	1.37 ± 0.22^b^

*Different superscripted letters within each line indicate significant differences (P < 0.05)*.

### Comparative Transcriptome Analysis

#### Differential Gene Statistics

**Figure 2:**
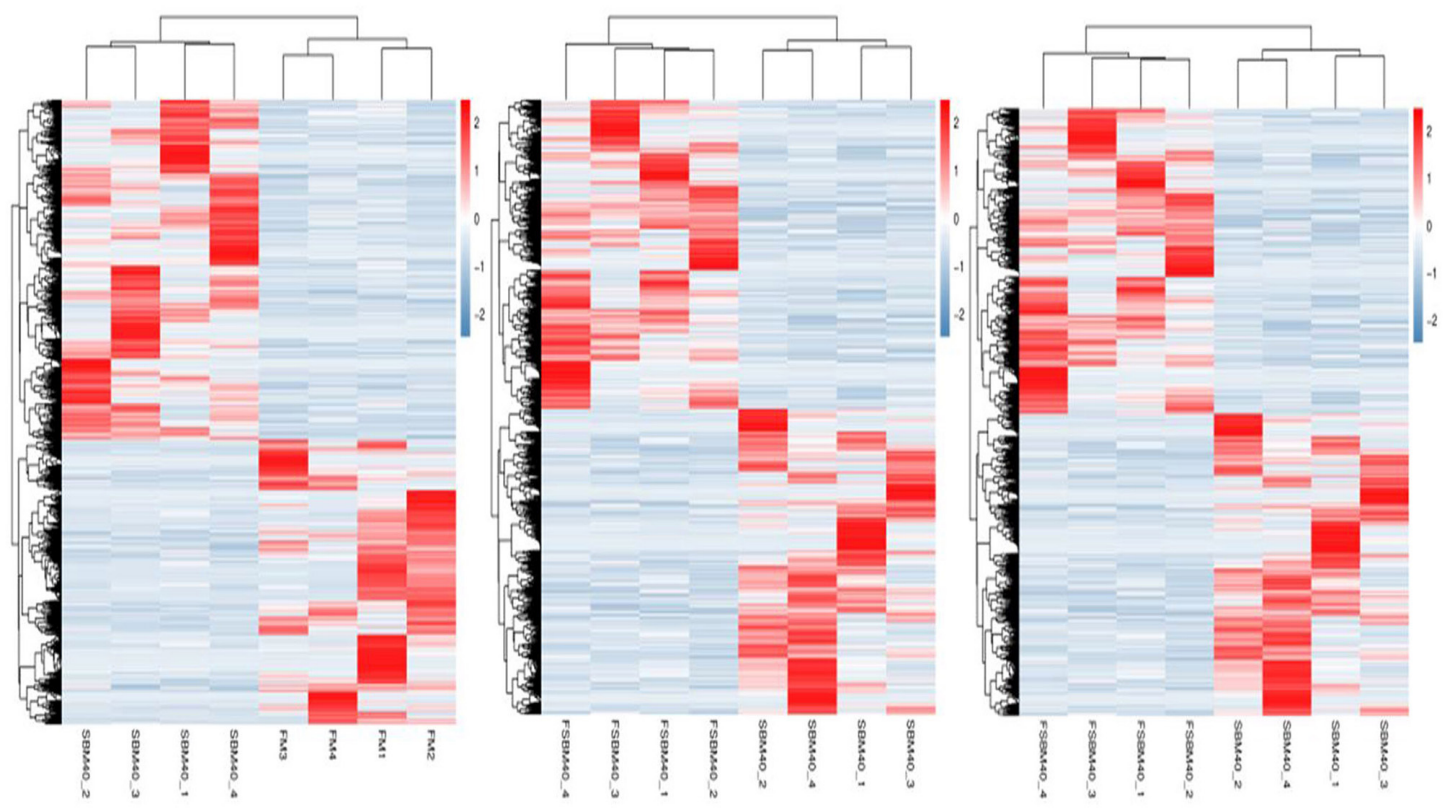
Cluster heat map of significantly differential genes in hindgut of pearl gentian grouper at different substitution levels of fermented soybean meal (*n* = 4).

**Figure 3:**
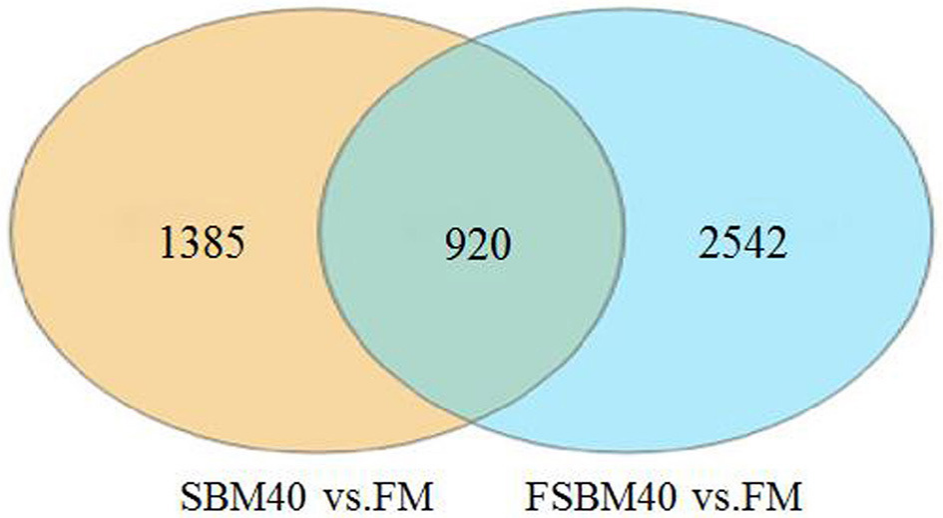
Venn map analysis of significant differential gene between soybean meal and fermented soybean meal in hindgut of pearl gentian grouper (*n* = 4).

The statistical results of DEGs are shown in Table 9. A total of 2,305 DEGs were identified in the SBM40 group, among which 1,256 were significantly upregulated (*P* < 0.05), and 1,049 were significantly downregulated (*P* < 0.05). Similarly, a total of 3,462 DEGs were identified in the FSBM40 group, among which 2,005 were significantly upregulated (*P* < 0.05), and 1,457 were significantly downregulated (*P* < 0.05). The cluster heatmap showed that the DEGs could cluster well-according to the group, indicating that the sample processing was reasonable and the quality control of the data was good (Figure 2). The Venn diagram of the DEGs (Figure 3) showed that there were 920 overlapping DEGs in the FSBM40 and SBM40 groups, named “Profile A”; there were 1,385 DEGs in the SBM40 group, named “Profile B”; and there were 2,542 unique DEGs in the FSBM40 group, named “Profile C.” As shown in the Venn plot, only 18.98% (920/5,445) of the DEGs had similar expression patterns, indicating that high levels of dietary SBM and FSBM have different metabolic mechanisms.

**Table 9:** Comparison of significant differential genes between fermented soybean meal and soybean meal substitute for fish meal in hindgut of pearl gentian grouper (*n* = 4).

**Number**	**SBM40 vs. FM**	**FSBM40 vs. FM**
Up	1,256	2,005
Down	1,049	1,457
Total	2,305	3,462

#### GO Enrichment Analysis

**Figure 4:**
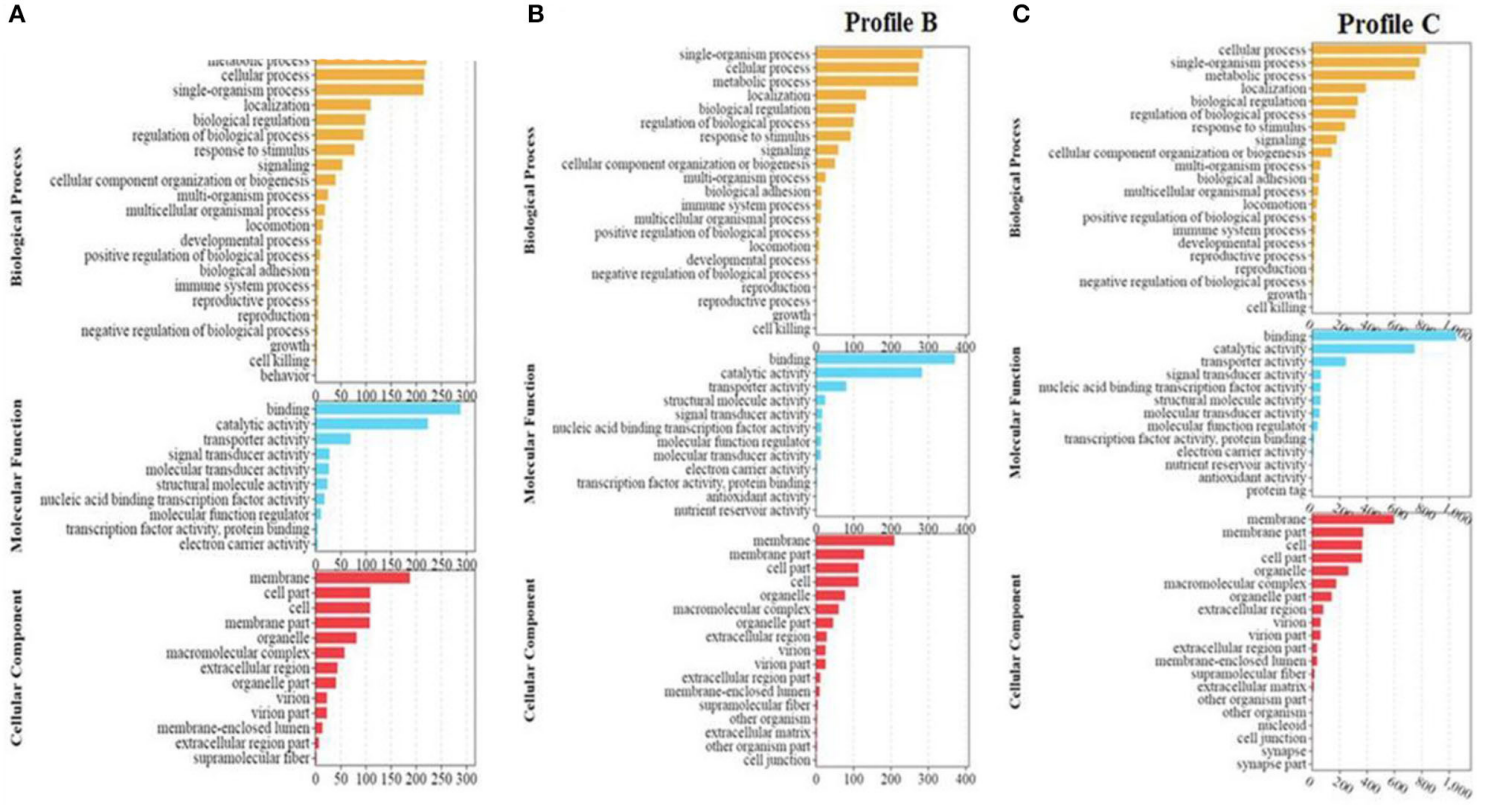
**(A–C)** GO enrichment analysis of significant differential genes between soybean meal and fermented soybean meal in hindgut of pearl gentian grouper (*n* = 4).

The enrichment analysis of Profile A showed that the DEGs were enriched in 45 subcategories in biological process, molecular function, and cellular component. In the subcategories of biological process, metabolic process (221) was the most enriched group, followed by cellular process (217), and single-organism process (215). In the subcategories of molecular function, binding (289) was the most enriched group, followed by catalytic activity (224). In the subcategories of cellular components, membrane (188) was the most enriched group, followed by cell (109), cell part (109), and membrane part (108) (Figure 4A).

Profile B was enriched into 50 sub-categories of the three primary categories mentioned above. In the subcategories of biological process, the metabolic process (222) was the most enriched group, followed by single-organism process (286), cellular process (276), and metabolic process (273). In the subcategories of molecular function, binding (372) and catalytic activity (284) were the most dominant groups. In the sub-categories of cellular components, membrane (210) was the most dominant group, followed by membrane part (129), and cell (114) (Figure 4B).

Profile C was enriched into 55 subcategories of the three primary categories mentioned above. In the subcategories of biological process, cellular process (835) was the most enriched group, followed by single-organism process (787), and metabolic process (754). In the subcategories of molecular function, binding (1,055) and catalytic activity (750) were the most enriched groups. In the subcategories of cellular components, membrane (599) was the most dominant group, followed by membrane part (374), and cell (365) (Figure 4C).

#### KEGG Enrichment Analysis

**Figure 5:**
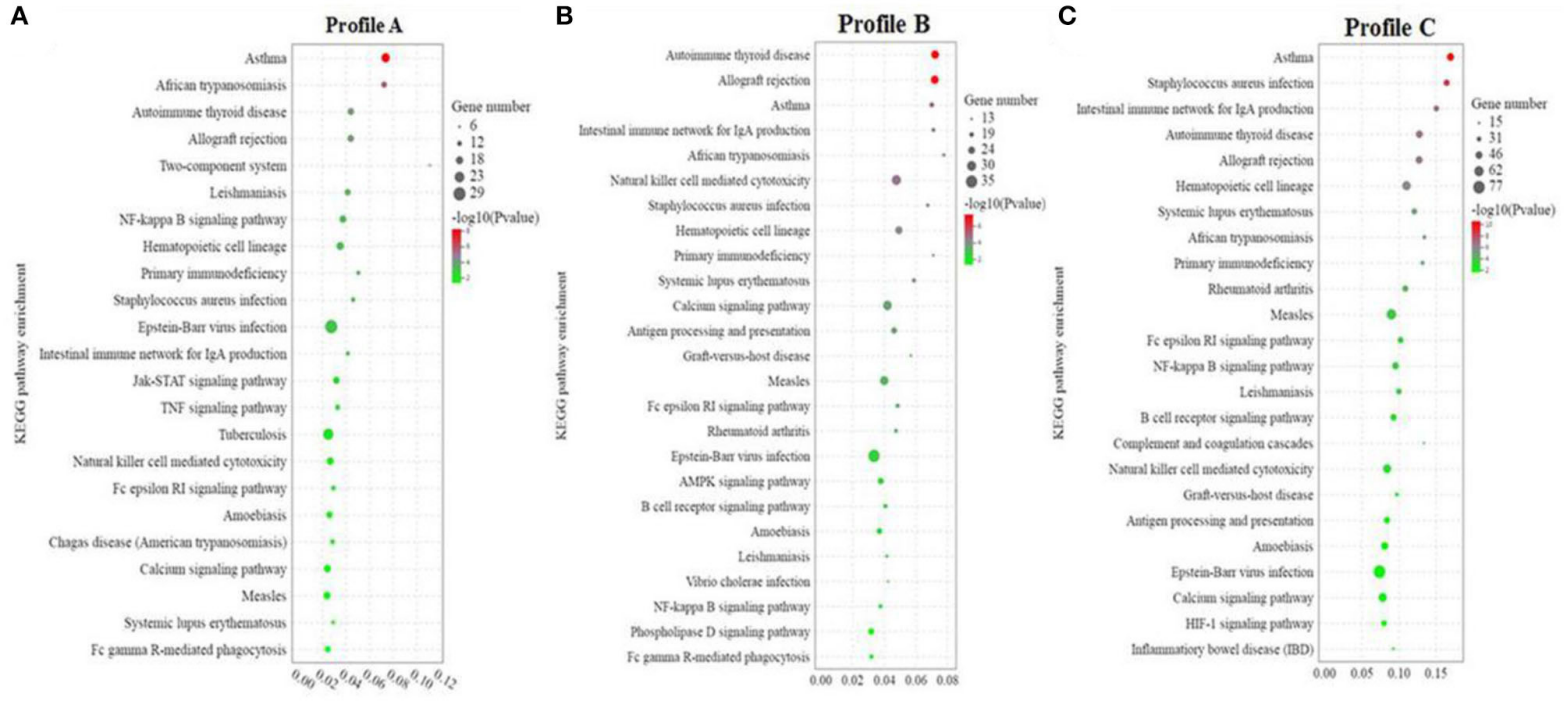
**(A–C)** KEGG enrichment analysis of significant differential genes between soybean meal and fermented soybean meal in hindgut of pearl gentian grouper (*n* = 4).

KEGG enrichment analysis was carried out on Profiles A, B, and C, respectively. Profile A enrichment results showed that 287 signaling pathways were enriched, 54 of which were significantly changed (*P* < 0.05). Among the pathways, 78 were related to immune diseases/systems, infectious diseases, and signal transduction, among which, 23 pathways were significantly enriched (*P* < 0.05). This indicated that among the enriched pathways, 42.59% (23/54) were related to immune diseases/system, infectious diseases, and signal transduction, including asthma, African trypanosomiasis, allograft rejection, two-component system, NF-κB signaling pathway, hematopoietic cell lineage, primary immunodeficiency, Epstein-Barr virus infection, intestinal immune network for IgA production, Jak-STAT signaling pathway, TNF signaling pathway, calcium signaling pathway, and Fc epsilon RI signaling pathway (Figure 5A).

Profile B enrichment results showed that 305 signaling pathways were enriched, 53 of which were significantly changed (*P* < 0.05). Among the pathways, 78 were related to immune diseases/systems, infectious diseases, and signal transduction, and 25 pathways were significantly enriched (*P* < 0.05). This indicated that among the enriched pathways, 47.17% (25/54) were related to immune diseases/systems, infectious diseases, and signal transduction, including autoimmune thyroid disease, allograft rejection, asthma, intestinal immune network for IgA production, African trypanosomiasis, natural killer cell mediated cytotoxicity, *Staphylococcus aureus* infection, primary immunodeficiency, calcium signaling pathway, AMPK signaling pathway, B cell receptor signaling pathway, *Vibrio cholerae* infection, NF-κB signaling pathway, Phospholipase D signaling pathway, and Fc epsilon RI signaling pathway (Figure 5B).

Profile C enrichment results showed that 335 signaling pathways were enriched, 57 of which were significantly changed (*P* < 0.05). Among the pathways, 81 were related to immune diseases/systems, infectious diseases, and signal transduction, and 24 pathways were significantly enriched (*P* < 0.05). This indicated that among the enriched pathways, 42.11% (24/57) were related to immune diseases/system, infectious diseases and signal transduction, including Asthma, *Staphylococcus aureus* infection, intestinal immune network for IgA production, autoimmune thyroid disease, allograft rejection, hematopoietic cell lineage, systemic lupus erythematosus, African trypanosomiasis, primary immunodeficiency, Fc epsilon RI signaling pathway, NF-κB signaling pathway, B cell receptor signaling pathway, complement and coagulation cascades, natural killer cell mediated cytotoxicity, antigen processing and presentation, calcium signaling pathway, and inflammatory bowel disease (IBD) (Figure 5C).

#### Validation of the Transcriptome Data by qRT-PCR

**Figure 6:**
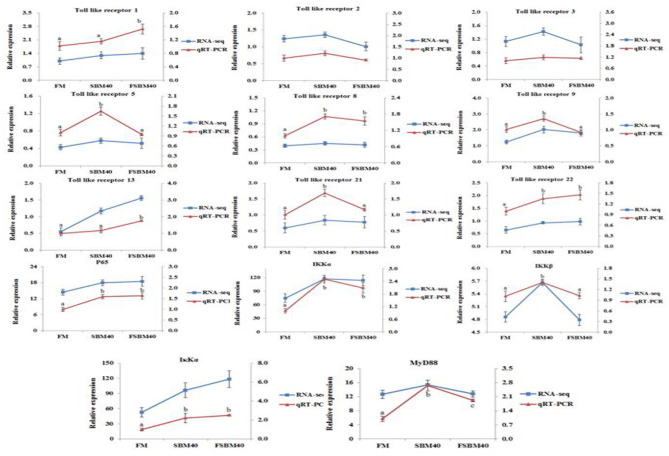
Comparison of RNA Seq and qRT-PCR results (*n* = 4). In order to validate RNA-seq data, qRT-PCR was used to detect the gene expression level of TLR-myD88-NF-κB pathway in DI tissue of pearl gentian grouper. The mRNA expression level of qRT-PCR was normalized by β-actin. The relative expression level in RNA-seq analysis was calculated by FPKM value. The statistical results were expressed as mean ± SD. Different letters assigned to the lines represented significantly differences between the groups at *P* < 0.05. FM, fish meal control group; FSBM40, 40% FSBM protein replacement level to FM protein; SBM40, 40% SBM protein replacement level to FM protein.

To verify the accuracy of transcriptome data generated using the “3+2” sequencing technology, we selected 14 toll-like receptor signal transduction and NF-κB signaling pathway genes related to immune and inflammatory development for qRT-PCR. Overall, the results of transcriptome sequencing were consistent with those of qRT-PCR, indicating that the transcriptome sequencing results were accurate. This confirmed the reliability of “3+2” transcriptome sequencing method (Figure 6).

## Discussion

The present study found that the growth performance, intestinal morphology, and immune-related gene expression were significantly affected by dietary FSBM. Previous studies indicated that the substitution effect of FSBM might be different for different culture subjects. In the hybrid tilapia diet, the optimum amount of FSBM substitution for FM was 34.2%; more than 60% substitution significantly affected its growth performance (Cheng and Liu, 2004). FSBM can substitute for 100% FM in *Ictalurus punctatus* diet, but the optimum substitution level was 25% (Li et al., 2007). An FSBM substitution level of more than 33.33% significantly affected the growth performance and feed utilization of *Litopenaeus vannamei* (Yang and Fu, 2008). Substituting 30% FM with FSBM (50% basal FM) in *Acanthopagrus latus* diet had no significant effects on FBW, SGR, and FCR (Ehsani et al., 2014). Wang (2009) reported that substituting 10% or lower of FM with FSBM improved the growth and apparent digestibility of *Micropterus salmoides*. The present study found that fish in the FSBM20 and FSBM40 groups had significantly lower WGR and SGR than those in the FM control. A previous study on *E. coioides* showed that a 14% dietary FSBM did not significantly affect WGR and SGR values; however, at higher levels, WGR and SGR values decreased significantly. The optimal amount of FSBM substitution was 10% (52% basal FM) (Luo et al., 2004). Compared with our unpublished results on the SBM feeding trial, the present study showed that FSBM at 20 and 40% substitution levels tended to improve the growth performance of the pearl gentian grouper; however, this was not significant. This may be attributed to the presence some ANFs even after processing by fermentation, or the short study period.

The intestinal tract is not only an important organ for digestion and absorption of nutrients, but also plays an important role in immune regulation, mucosal barrier, signal recognition, and endogenous active molecule production (Cerezuela et al., 2013). Moreover, intestinal structure and functions are sensitive to changes in the quality and quantity of dietary nutrition, especially in the state of nutritional deficiency (Wang et al., 2017). Currently, there are few studies on the effect of dietary FSBM on the intestinal morphology of fish (He et al., 2014). Research on *Larimichthys crocea* found that 15–75% substitution of FM with FSBM had no significant effect on intestinal structure, induced liver lesions at higher substation levels (Feng et al., 2016). At the histological level, the present study found that dietary FSBM induced changes in the intestinal morphology of the pearl gentian grouper, such as lower plica, wider lamina propria, shorter microvilli, and increased inflammatory infiltration. The differences in results of studies on FSBM may be due to the use of different breeds of fish. Previous research has reported that SBMIE is characterized by reduced mucosal fold height, swelling of lamina propria and submucosa, loss of absorption vacuolation in the normal intestinal nucleus, and increase in the concentration of inflammatory cells, resulting in a decrease in the digestive and nutritional capacity of DI tissue (Gu et al., 2018). Similar results were found in the present experiment, indicating that the substitution of FM with FSBM induced enteritis in the pearl gentian grouper.

The effects of FSBM on the intestinal health of the pearl gentian grouper were reflected in variations in enzyme activities in the DI tissues. The present study found that Try enzyme activity significantly increased with dietary FSBM addition. Related research has shown that a characteristic of fish SBMIE is the increase in Try enzyme activity in DI tissues (Lilleeng et al., 2007). Fish immune status is largely dependent on humoral and cellular immunity. Humoral immunity of fish includes non-specific immunity and specific immunity. Complement and LYS are important components of non-specific immunity, and IgM is an important component of specific humoral immunity. C3 and C4 play key roles in the activation of the complement system (Watts et al., 1995; Kuroda et al., 2000). The present study found that the concentrations of C3, C4, and IgM decreased with an increase in dietary FSBM and this may be because FSBM impaired the intestinal immune function of the pearl gentian grouper. MDA, a lipid peroxide, is one of the final products of oxidative stress, resulting from the breakdown PUFA in biofilms by oxygen free radicals. The concentrations of MDA in animal tissues indicate the rate or intensity of lipid peroxidation in tissues and cells (Wen et al., 2015). In the present study, the concentration of MDA in the DI tissue significantly increased with an increase in dietary FSBM, indicating that dietary FSBM caused intestinal injury in the pearl gentian grouper.

Based on immune responses, cytokines are divided into pro-inflammatory cytokines and anti-inflammatory cytokines. It has been reported that SBMIE in fish is accompanied by upregulation of the expression of pro-inflammatory genes and downregulation of the expression of anti-inflammatory genes (Sahlmann et al., 2013; Wang et al., 2017). Similar results were found in this study. The expression of pro-inflammatory factors, such as *IL1*β, *IL8, IL17 TNF*α, and *CSF1* increased significantly with an increase in dietary FSBM, while the expression of anti-inflammatory genes, such as *IL4, IL10*, and *TGF*β*1* decreased significantly with an increase in dietary FSBM. The increase in the expression of anti-inflammatory genes may inhibit the expression of tight junction protein genes. Alsadi et al. (2009) reported that an increase in most anti-inflammatory factors, such as *IL1*β, *IFN-*γ*2*, and *TNF*α, could lead to the destruction of tight junction barrier of epithelial cells. Pan et al. (2017) reported that the downregulation of the expression of inflammatory genes, *IL-1*β, *IL6, IL8, IL15, IL-17D, IFN-*γ*2*, and *TNF*α was negatively correlated with the expression genes (*claudin-3, -b, -c, occludin*, and *ZO-1*) regulating the biosynthesis of the tight junction protein. The effect of FSBM on the expression of genes regulating the biosynthesis of the tight junction proteins in the intestine of the pearl gentian grouper needs further study.

Our previous study found that SBMIE could also be induced by high levels of SBM (Zhang et al., 2021a). To understand the differential mechanism of enteritis induced by FSBM and SBM, a transcriptome comparison analysis was performed on the intestinal tissues of the pearl gentian grouper fed the two kinds of diets. Transcriptome analysis of the effects of replacing FM with fermented plant proteins on the intestinal health of fish has rarely been reported. Recently, leguminous plants and their concentrates, such as soybeans, peas, and lupins, have been frequently used in the formulation of fish feeds. Moreover, in the past, most of the omics studies on plant protein-induced enteritis in fish were carried out on Atlantic salmon (Martin and Król, 2017) using mainly DNA microarray technology; these studies identified some conservative genes and signal pathways. Kortner et al. (2012) reported that the combination of pea protein concentrate and soyasaponin can induce DI enteritis and immune gene expression variations in Atlantic salmon, such as the upregulation of inflammatory cytokines, NF-κB signaling pathway, TNF-α signaling pathway related genes, and T cell function regulators. Sahlmann et al. (2013) reported that SBM can cause DI enteritis at the tissue level in Atlantic salmon, and increase the expression of immune-related genes, including the GTPase IMAP family, NF-κB signaling pathway, IL8 signaling pathway, and regulatory factors of T cell and B cell, and then downregulate transcripts related to endocytosis, exocytosis, detoxification, transportation, and metabolism. De Santis et al. (2015) indicated that diets containing 30% SBM altered the expression of immune-related genes of DI tissues in Atlantic salmon, including phagocytosis, antigen processing, and presentation signaling pathways. In the present study, similar to SBM, enteritis induced by FSBM was enriched in immune-related signaling pathways, such as NF-κB signaling pathway, TNF signaling pathway, antigen processing and presentation, intestinal immune network producing IgA, inflammatory bowel disease (IBD), B cell receptor, and JAK-STAT. Furthermore, SBM and FSBM accounted for a similar proportion (both ~45%) of significantly affected pathways associated with immune diseases/systems, infectious diseases, and signal transduction. The reason for this phenomenon may be due to the incomplete removal ANFs, or an imbalance of intestinal flora in FSBM, which affected the intestinal health of fish and eventually induce enteritis.

Similar to SBM-induced enteritis, the present study found that the NF-κB signaling pathway, which is closely related to the development of inflammation, was conservative during the process of FSBM-induced enteritis in the pearl gentian grouper. Meanwhile, some studies have shown that intestinal flora also plays an important role in regulating the immune response of the intestinal mucosa (Miao et al., 2018). In humans and other animal, studies have confirmed the role of intestinal microorganisms in the pathogenesis of inflammatory bowel disease (IBD) (Marteau et al., 2003). Harper et al. (1985) reported that Crohn's disease patients treated with sterile ultrafiltration of small intestinal exudate did not show intestinal inflammation; however, the reentry of intestinal exudate led to enteritis. In this study, dietary FSBM significantly affected the intestinal flora of the pearl gentian grouper, which was confirmed by the significant variations in lysozyme (Aoki et al., 2002), indicating that the intestinal flora may play an important role in the process of FSBM-induced enteritis.

Pathogen infection can trigger the inflammatory response of host animals, and the host can sense the pathogen signal and initiate response through pattern recognition receptors (PRRs). Toll-like receptors are the most widely studied PRRs in mammals (Takeuchi and Akira, 2010). TLRs play an indispensable role in innate immune responses, which are the first line of defense against pathogen invasion. They play a key role in inflammation, immune cell regulation, survival, and proliferation. In a study of enteritis using sterile zebrafish as a model, it was found that the induction of enteritis depended on the transduction of intestinal flora and the toll-like receptor (TLR) signaling pathway (Oehlers et al., 2011). Researchers have examined the mechanism by which TLRs recognize symbiotic bacteria under normal conditions and pathogen-related molecular patterns (PAMPs), such as lipopolysaccharide, double-stranded RNA (ds RNA), and flagella, and promote a series of immune defense mechanisms. The interaction of TLRs with symbiotic bacterial products plays an important role in intestinal homeostasis and resistance to epithelial injury (Rakof-Nahoum et al., 2004). Dysfunctional interactions between bacteria and TLRs may contribute to chronic inflammation. The TLRs-NF-κB signaling pathway is the main component of inflammation and immune response in organisms (Medzhitov and Janeway, 2000; Tan et al., 2016). Based on the above analysis, the present study examined the role of the TLRs-NF-κB signaling pathway in the development of enteritis in pearl gentian grouper.

In the present study, nine TLRs, including TLR1, TLR2, TLR3, TLR5, TLR8, TLR9, TLR13, TLR21, and TLR22, were identified in the intestinal tissue of the pearl gentian grouper, using PacBio SMRT sequencing. The types of TLR present in intestinal tissues differ with fish species. Except TLR8, all TLRs identified in the present study have been reported in *E. coioides* (Wei et al., 2011; Ding et al., 2012; Li et al., 2012; Lin et al., 2013; Chen et al., 2015; Liang et al., 2018; He et al., 2019). TLR8 has been identified in channel catfish, grass carp, zebrafish (Sullivan et al., 2009; Huang et al., 2012; Quiniou et al., 2013). Currently, at least 20 TLRs have been identified in fish. Among the TLRs identified in the present study, TLR1, TLR2, TLR3, TLR5, TLR9, TLR21, and TLR22 have been reported in fish as sensors for bacterial ligands. Wei et al. (2011) reported that bacterial lipopolysaccharide and poly (I: C) could upregulate the expression of *TLR1* and *TLR2* in the spleen and head kidney of *E. coioides*. Similarly, the bacterial pathogen *Vibrio alginolyticus* can upregulate the expression of *TLR1* and *TLR2* in the spleen and head kidney. Tsujita et al. (2004) found that *Vibrio anguillarum* or its flagellum can activate the expression of *TLR5* in rainbow trout, and flagellin mediates the significant activation of NF-κB. The bacterial ligand of *TLR9* is the nucleic acid of bacteria or viruses rather than bacterial components. Byadgi et al. (2014) revealed that oligodeoxyribonucleotides containing CpG (CpG ODN) could significantly promote the expression of *TLR9* and pro-inflammatory factors in the liver and spleen of *Rachycentron canadum*. Yeh et al. (2013) discovered that *TLR9* and *TLR21* have similar expression profiles in zebrafish and co-mediate the antibacterial activity of CpG ODN. TLR22 is a unique receptor in fish and unlike TLR3, which is located in the endosome/lysosome to recognize short dsRNA, TLR22 is located on the cell surface to recognize long dsRNA (Matsuo et al., 2008). The present study showed that the expression levels of *TLR5, TLR8, TLR9, TLR21*, and *TLR22* were significantly increased after FM was replaced by SBM; while the replacement of FM with FSBM significantly increased the expression of *TLR1, TLR8, TLR13*, and *TLR22*, indicating that the intestinal pathogenic flora activated toll-like receptor signal transduction through a variety of bacterial components/products after SBM and FSBM replaced FM. However, there were some differences in the TLRs pathways used by fish fed the diets.

When TLRs are activated, they recruit relevant adaptor proteins in the cytoplasm and trigger different signaling cascades. The TLR signaling pathway can be divided into two types-the MyD88-dependent pathway and MyD88-independent pathway (TRIF-dependent) (Rauta et al., 2014). Intracellular components of the downstream TLR signaling pathway are highly conserved in mammals and teleosts (Purcell et al., 2006). In addition to TLR3, MyD88 can be recruited by all TLR members to activate the downstream NF-κB signaling pathway and the mitogen-activated protein kinase signaling pathway (O'Neill and Bowie, 2007; Kawai and Akira, 2010). However, in the MyD88-independent signaling pathway, TRIF can only be recruited by TLR3 and TLR4 to activate the NF-κB signaling pathway and transcription factor IFR3 (Kanwal et al., 2014). In the present study, *TLR4* was not detected in the pearl gentian grouper, and the expression level of *TLR3* did not significantly change with dietary FSBM or SBM. However, the expression levels of key genes (*IKK*α, *IKK*β, *I*κ*B*α, and *p65*) and *MyD88* in the NF-κB signaling pathway were significantly increased by increase in dietary FSBM and SBM, indicating that the TLR-MyD88-NF-κB signaling pathway plays an important role in FSBM- and SBM-induced enteritis in the pearl gentian grouper.

## Data Availability Statement

The datasets presented in this study can be found in online repositories. The names of the repository/repositories and accession number(s) can be found in the article/Supplementary Material.

## Ethics Statement

The animal study was reviewed and approved by the ethics review board of Guangdong Ocean University. All of the procedures were performed in accordance with the Declaration of Helsinki and relevant policies in China.

## Author Contributions

WZ designed and took part in the whole process of the experiment, and wrote the draft of this manuscript. BT and JD co-conceived the experiment, revised the draft critically for important intellectual content. XD and QY participated in the experiments and SC revised the first draft. HL and SZ analyzed the data. SX and HZ approved the final version. All authors contributed to the article and approved the submitted version.

## Conflict of Interest

The authors declare that the research was conducted in the absence of any commercial or financial relationships that could be construed as a potential conflict of interest.
