# Supplementary material for: Mechanisms by Which Fermented Soybean Meal and Soybean Meal Induced Enteritis in Marine Fish Juvenile Pearl Gentian Grouper
Source: Front Physiol. 2021 Apr 22;12:646853. doi: 10.3389/fphys.2021.646853 (PMC8100241; doi:10.3389/fphys.2021.646853)
Supplement: Supplementary file 1 [file Table_1.DOCX]

**Supplementary Table 1** Formulation and proximate composition of the SBM experimental diets (%, dry matter)

| Ingredients (%) | Diets | | |
| --- | --- | --- | --- |
|  | FM | SBM20 | SBM40 |
| Red fish meal | 50.00 | 40.00 | 30.00 |
| Soybean meal | 0.00 | 14.83 | 29.65 |
| Vital wheat gluten | 5.00 | 5.00 | 5.00 |
| Wheat flour | 18.00 | 18.00 | 18.00 |
| Casein | 4.60 | 4.60 | 4.60 |
| Gelatin | 1.00 | 1.00 | 1.00 |
| Fish oil | 3.02 | 3.75 | 4.48 |
| Soybean oil | 2.00 | 2.00 | 2.00 |
| Soybean lecithin | 2.00 | 2.00 | 2.00 |
| Microcrystalline cellulose | 11.48 | 5.74 | 0.00 |
| Calcium monophosphate | 1.50 | 1.50 | 1.50 |
| Ascorbic acid | 0.05 | 0.05 | 0.05 |
| Choline chloride | 0.50 | 0.50 | 0.50 |
| Vitamin premix^a^ | 0.30 | 0.30 | 0.30 |
| Mineral premix^b^ | 0.50 | 0.50 | 0.50 |
| Ethoxyquin | 0.05 | 0.05 | 0.05 |
| Lysine^c^ | 0.00 | 0.12 | 0.24 |
| Methionine^c^ | 0.00 | 0.06 | 0.13 |
| Proximate composition) (%, dry matter) | | | |
| Crude protein | 50.97 | 50.56 | 50.85 |
| Crude lipid | 10.15 | 10.50 | 10.44 |

Note: Same as Table 1.
