# Supplementary material for: Mechanisms by Which Fermented Soybean Meal and Soybean Meal Induced Enteritis in Marine Fish Juvenile Pearl Gentian Grouper
Source: Front Physiol. 2021 Apr 22;12:646853. doi: 10.3389/fphys.2021.646853 (PMC8100241; doi:10.3389/fphys.2021.646853)
Supplement: Supplementary file 2 [file Table_2.DOCX]

**Supplementary Table 2** The content of 17 amino acids in experimental diets (%)

| Amino acids | Diets | | | | |
| --- | --- | --- | --- | --- | --- |
|  | FM | SBM20 | SBM40 | FSBM20 | FSBM40 |
| Lysine | 3.02 | 2.93 | 2.88 | 2.89 | 2.83 |
| Methionine | 1.01 | 0.95 | 0.86 | 1.00 | 1.02 |
| Arginine | 2.53 | 2.43 | 2.47 | 2.35 | 2.39 |
| Threonine | 1.78 | 1.75 | 1.73 | 1.76 | 1.75 |
| Isoleucine | 1.86 | 1.78 | 1.82 | 1.81 | 1.84 |
| Histidine | 1.28 | 1.27 | 1.20 | 1.25 | 1.25 |
| Valine | 2.10 | 2.00 | 1.98 | 2.01 | 2.04 |
| Leucine | 3.28 | 3.16 | 3.20 | 3.21 | 3.22 |
| Phenylalanine | 1.94 | 1.88 | 1.96 | 1.89 | 1.93 |
| Tyrosine | 1.47 | 1.38 | 1.43 | 1.37 | 1.39 |
| Aspartate | 3.86 | 3.78 | 3.91 | 3.78 | 3.83 |
| Serine | 1.89 | 1.87 | 1.97 | 1.89 | 1.89 |
| Glutamate | 8.17 | 7.83 | 8.33 | 7.92 | 8.13 |
| Glycine | 2.48 | 2.42 | 2.99 | 2.41 | 2.40 |
| Alanine | 2.37 | 2.33 | 2.20 | 2.34 | 2.31 |
| Proline | 2.78 | 2.61 | 2.82 | 2.77 | 2.81 |
| Cystine | 0.49 | 0.49 | 0.51 | 0.50 | 0.50 |
