# Supplementary material for: Mechanisms by Which Fermented Soybean Meal and Soybean Meal Induced Enteritis in Marine Fish Juvenile Pearl Gentian Grouper
Source: Front Physiol. 2021 Apr 22;12:646853. doi: 10.3389/fphys.2021.646853 (PMC8100241; doi:10.3389/fphys.2021.646853)
Supplement: Supplementary file 3 [file Table_3.DOCX]

**Supplementary Table 3** Determination of anti-nutritional factors in FSBM and SBM diets（mg/kg）

| Group | FM | SBM20 | SBM40 | FSBM20 | FSBM40 |
| --- | --- | --- | --- | --- | --- |
| Glycitin | 0 | 41.09 | 46.13 | 21.44 | 23.5 |
| Genistin | 0 | 59.46 | 93.19 | 45.37 | 95.41 |
| Glycitein | 0 | 0 | 26.17 | 0 | 21.42 |
| Genistein | 0 | 49.06 | 82.36 | 20.68 | 38.26 |
| Soyasaponin I | 0 | 2121.21 | 3082.41 | 2402.17 | 3214.22 |
| Daidzein | 0 | 162.26 | 302.79 | 133.58 | 233.45 |
| Soybean 7S globulin | 0 | 32421.06 | 42586.41 | 43749.75 | 50179.81 |
| Soybean 11S globulin | 0 | 33556.66 | 48911.94 | 45821.29 | 52368.87 |
