# Supplementary material for: Mechanisms by Which Fermented Soybean Meal and Soybean Meal Induced Enteritis in Marine Fish Juvenile Pearl Gentian Grouper
Source: Front Physiol. 2021 Apr 22;12:646853. doi: 10.3389/fphys.2021.646853 (PMC8100241; doi:10.3389/fphys.2021.646853)
Supplement: Supplementary file 4 [file Table_4.DOCX]

**Supplementary Table 4** Semi-quantitative evaluation criteria for the morphology of intestinal histology of enteritis

induced by soy meal of pearl gentian grouper

| Descriptive parameter | Score 1 | Score 2 | Score 3 | Score 4 | Score 5 |
| --- | --- | --- | --- | --- | --- |
| Mucosal folds | simple and complex plica, long and slender | moderate simple plica length, thicker complex plica | simple plica short to medium length, lateral branches complex plica short and thick | simple plica short and thick, thick and short complex plica ubiquitous | both simple and complex plica very short shape |
| Lamina propria | very thin lamina propria, fragile core of connective tissue | lamina propria slightly widened in some plica | lamina propria obviously widening in most plica | lamina propria thickened in many plica | very thick lamina propria in many plica |
| Supranuclear vacuoles | large vacuoles, almost occupying entire top of intestinal cells | medium sized vacuoles occupying less than half of the intestinal cells | small vacuoles near the apical membrane in most intestinal cells | small vacuoles still dispersing in some intestinal cells | no nuclear vacuoles |
| Connective tissue | almost no connective tissue between the base of plica and dense layer | connective tissue quantity under the plica slightly increasing | mucous plica significantly increasing beneath most connective tissue | thick connective tissue beneath many plica | a very thick layer of connective tissue beneath some plica |
