# Supplementary material for: Mechanisms by Which Fermented Soybean Meal and Soybean Meal Induced Enteritis in Marine Fish Juvenile Pearl Gentian Grouper
Source: Front Physiol. 2021 Apr 22;12:646853. doi: 10.3389/fphys.2021.646853 (PMC8100241; doi:10.3389/fphys.2021.646853)
Supplement: Supplementary file 5 [file Table_5.DOCX]

**Supplementary Table 5** Effect of different levels of soybean meal protein substitute for fish meal protein on the growth of pearl gentian grouper (n=3)

| Parameters | FM | SBM20 | SBM40 |
| --- | --- | --- | --- |
| IBW(g) | 12.55±0.00 | 12.55±0.01 | 12.55±0.04 |
| WGR(%) | 485.14±7.08^a^ | 464.36±10.12^b^ | 426.50±9.59^c^ |
| SGR(%d) | 2.60±0.02^a^ | 2.54±0.03^b^ | 2.44±0.03^c^ |
| FCR | 0.84±0.01^a^ | 0.87±0.02^b^ | 0.95±0.02^c^ |
| HSI(%) | 2.43±0.45 | 2.21±0.36 | 2.18±0.25 |
| SR(%) | 99.17±0.96 | 99.58±0.84 | 99.17±0.96 |

Note: The results are presented as the means *±* SD. Values in a row with different superscripts represented significantly different at *P* < 0.05. IBW, initial body weight; FBW, final body weight; WGR, weight gain rate; SGR, special growth rate,; FCR, feed conversion rate; HSI, hepatosomatic index; SR, survival rate.
